# Management of dermatologic adverse events from cancer therapies: recommendations of an expert panel^☆^^☆☆^

**DOI:** 10.1016/j.abd.2020.01.001

**Published:** 2020-02-15

**Authors:** Jade Cury-Martins, Adriana Pessoa Mendes Eris, Cristina Martinez Zugaib Abdalla, Giselle de Barros Silva, Veronica Paula Torel de Moura, Jose Antonio Sanches

**Affiliations:** a Department of Dermatology, Hospital das Clínicas, Faculdade de Medicina, Universidade de São Paulo, São Paulo, SP, Brazil; b Dermatology Clinic, Santa Casa de Misericórdia de São Paulo, São Paulo, SP, Brazil; c Skin Cancer Unit, A.C. Camargo Cancer Center, São Paulo, SP, Brazil; d Dermatology Unit, Hospital Sírio-Libanês, São Paulo, SP, Brazil; e Dermatology Unit, Centro Paulista de Oncologia, São Paulo, SP, Brazil; f Oncology Center, Hospital Beneficência Portuguesa de São Paulo, São Paulo, SP, Brazil

**Keywords:** Antineoplastic agents, Antineoplastic agents, immunological, Dermatology, Drug-related side effects and adverse reactions, Medical oncology, Molecular targeted therapy

## Abstract

With the development of new cancer therapies, systemic toxicity profile and effects on survival achieved an important improvement. However, a constellation of toxicities has emerged, even more remarkably, cutaneous adverse events. This report, developed by a board of Brazilian experts in oncodermatology, aims to establish a guideline for the dermatological care of oncologic patients. When possible, evidence-based recommendations were made, but in many cases, when strong evidence was not available, a consensus was reached, based on some data supporting therapies combined with personal experiences.

## Introduction

With the advance of cancer treatment, systemic toxicities and survival faced an important improvement. However, its use is often related to dermatologic adverse events (DAE), which have a high frequency, are often symptomatic, may be disfiguring, and might cause an important impact on patient's quality of life (QoL). Another important issue relies on the fact that those skin toxicities might lead to dose reductions or even discontinuation of cancer therapy, with impact on the disease outcome. For those reasons, it is important for dermatologists to know the most common types of reactions in order to be able to help patients and oncologists on the prevention and management of those toxicities.^1^, ^2^

A multidisciplinary team with a good interaction between the different specialists (e.g.: oncologists, dermatologists, nurses) is fundamental for the best supportive care for cancer patients and their families. Different societies and associations are dedicated to the research, support and education in all aspects of cancer treatment, such as the Multinational Association of Supportive Care in Cancer (MASCC), the American Society of Clinical Oncology (ASCO), the Oncology Nurse Society (ONS), and the National Comprehensive Cancer Network (NCCN).

The aim of this paper was to establish a guideline to help professionals on the dermatological care of oncologic patients. When possible, evidence-based recommendations were made, but when strong evidence was not available, a consensus was reached based on some data supporting therapies combined with personal experiences. Levels of evidence are defined bellow and are reported for each treatment in Table 1, Table 2, Table 3.

**Table 1:** Dermatologic adverse events of conventional chemotherapeutic agents.

Dermatologic toxicity	Most frequent agents	Management	Level of evidence
**Hyperpigmentation**		-Photoprotective measures -Bleaching agents	IV IV
Diffuse	Busulfan (“tan” appearance); methotrexate; procarbazine, capecitabine		
Flagellate	Bleomycin		
Serpentine supravenous	5-FU, docetaxel, vincristine, vinorelbine		
Areas of pressure, flexural, under occlusive dressings	Ifosfamide, thiotepa, hydroxyurea, cisplatin, docetaxel		
Palms and soles	Hydroxyurea, doxorubicin, capecitabine		
Palmar creases	Ifosfamide, cyclophosphamide bleomycin, busulfan, doxorubicin		
Mucous membranes	Cyclophosphamide (gingiva), busulfan, doxorubicin, cisplatin, capecitabine		
**Nail changes**		*General measures to all patients*: reduction of contact to water, use of cotton gloves beneath plastic or rubber gloves for any wet work, avoidance of any damaging procedure (e.g.: aggressive manicuring), and hydration with thick emollients	IV
Onycholysis	Taxanes, cyclophosphamide doxorubicin, capecitabine, etoposide	*Prevention*: frozen gloves and socks during infusion (depending on the agent and type of infusion)	IIA
		*Treatment:*	
		-Avoiding traumas (e.g.: keeping nails short) and humidity	IV
		-If green discoloration: topical antibiotic (tobramycin or ciprofloxacin eye drops)	IV
Paronychia	Taxanes, etoposide, capecitabine	-Topical corticosteroids -If secondary infection is suspected, culture guided antibiotics	IV IB
Nail pigmentation	5-FU, taxanes, hydroxyurea, cyclophosphamide, doxorubicin, capecitabine	Might be diffuse or banding/streaking. No treatment needed.	
**Hand-foot syndrome (HFS)**	Capecitabine, doxorubicin, citarabine, 5-FU, taxanes	*Prevention:*	
		-regional cooling during chemotherapy infusion	IB
		-nonsteroidal anti-inflammatory agent	IA
		-use thick cotton gloves and/or socks; urea based emollients; avoid irritants and tight clothing and shoes; avoid extremes of temperature, pressure and friction	III
		*Treatment:*	
		-potent topical steroid	III-IV
		-for relief of symptoms, cool compresses or emergence of hands and feet on cool water, topical anesthetics and NSAIDs	III-IV
		-Dose reduction or treatment interruption is sometimes necessary	III-IV
**PATEO syndrome**	Docetaxel	See measures for HFS above	
**Chemotherapy-induced alopecia**	Cyclophosphamide, doxorubicin, irinotecan and taxanes	*Prevention*: scalp cooling devices	I
		*Treatment:*	
		-Topical Minoxidil 2%	IB
		-Topical minoxidil 5%, biotin containing oral supplements	IV
		-Topical bimatoprost (for eyelashes)	IIA

5-FU, 5-Ffluouracil; NSAID; nonsteroidal anti-inflammatory drug; PATEO, PeriArticular Thenar Erythema and Onycholysis.

**Table 2:** Dermatologic adverse events of targeted therapies.

Drug class	Agents	Dermatologic toxicities	Management	Level of evidence
**EGFR inhibitors**	-Monoclonal antibodies: cetuximab and panitumumab -TKI specific for EGFR: erlotinib and gefitinib -Less specific multikinase inhibitors: vandetanib	Papulopustular eruption (starts on the first 2 weeks)	*Prevention:*	
			-Systemic antibiotics for the first 6–8 weeks (tetracyclines), sunscreen	IB
			*Treatment:*	
			-Low potency topical steroids	III
			-Systemic antibiotics (tetracyclines)	IB
			-Systemic isotretinoin (low doses)	III
			-Culture-driven antibiotics if secondary infection	IB
		Xerosis	-Limited shower time, use of gentle cleansers (pH-neutral soaps or syndets), regular use of emollients	III
			-Topical steroid if eczematous lesions	III
		Paronychia and pyogenic granuloma like lesions	*Prevention:*	
			-Systemic antibiotics (tetracyclines)	IB
			-Antiseptic solutions	III
			*Treatment:*	
			-Topical steroids	III
			-Systemic antibiotics (tetracyclines)	III
			-Culture-driven antibiotics if secondary infection	IB
		Fissures	Protective coverings (hydrocolloid, biological or cyanoacrylate glue), barrier creams (petroleum jelly, zinc oxide cream) and thick emollients	IIB
		Hair changes	-Nonscarring alopecia: topical minoxidil	IB
			-Inflammatory and scarring alopecia: topical steroids	III
			-Trichomegaly: eyelash trimming	III
			-Hypertrichosis: laser hair reduction	IB
**KIT and BCR-ABL inhibitors**	Imatinib, nilotinib, dasatinib	Exanthema (rash)	Topical steroids or short courses of oral steroids	III
		Hypopigmentation	Reversible after treatment interruption	III
**Antiangiogenic agents**	-Selective VEGFR inhibitors: bevacizumab and ranibizumab Non-selective multikinase inhibitors: sorafenib, pazopanib, sunitinib	Hand-foot skin reaction	*Prevention:*	
			-Use thick cotton gloves and/or socks; urea based emollients; avoid irritants and tight clothing and shoes; avoid extremes of temperature, pressure and friction	III
			-Pretreatment evaluation with a podiatrist with callosity chopping and the use of orthopedic shoe inserts when needed	III
			-Urea based emollients	IB
			*Treatment:*	
			-Keratolytic agents	III
			-Topical corticosteroids	III
			-Potent topical steroid	III
			-For relief of symptoms, cool compresses or emergence of hands and feet on cool water, topical anesthetics and NSAIDs	III
			-Hydrocolloid dressings?	IB
		Pigmentary changes	-Hypopigmentation of hair and skin (pazopanib and sunitinib), yellow discoloration of skin (sunitinib) – reversible after discontinuation	III
		Hair and scalp	-Seborrheic dermatitis-like rash: topical steroids	III
			-Non-scarring alopecia: topical minoxidil	IV
**RAF inhibitors**	Vemurafenib and dabrafenib	Exanthema (rash)	-Oral antihistamines, topical or short courses of systemic steroids	III
			*Temporary treatment interruption might be necessary	
		Photosensitivity	*Prevention:* photoprotective measures	IIB
			*Treatment:* topical or short courses of systemic steroids	III
			*Mostly vemurafenib, UVA-induced	
		Keratosis pilaris like eruption	Keratolytics and emollients, gentle skin care	III
		Seborrheic dermatitis-like	Topical steroids	III
		Hand-foot skin reaction	See above (antiangiogenic agents)	
		Keratoacanthomas and squamous cell carcinomas	-Frequent dermatological monitoring	III
			-If few lesions: surgical excision	III
			-If multiple lesions: 5-FU, systemic retinoids or photodynamic therapy	IIA/B
			*Association with a MEKi decreases lesions	
		Warts and verrucal keratoses	-Destructive or surgical measures	III
			-Topical treatments: keratolytics, 5-FU, imiquimod	III
**MEK inhibitors**	Cobimetinibe, trametinibe, selumetinibe	Papulopustular eruption	See EGFR inhibitors above	
		Xerosis		
		Paronychia		
		Exanthema (rash)	-Oral antihistamines, topical or short courses of systemic steroids	III
mTOR inhibitors	Rapamycin, everolimus, sirolimus	Stomatitis	Antiseptic washes, topical steroids and anesthetics	IV

* NSAID, nonsteroidal anti-inflammatory drug.

**Table 3:** Dermatologic adverse events of immunotherapy (immune-related adverse events – irAE).

Drug class	Agents*	Dermatologic toxicities	Management	Level of evidence
**“Checkpoint” inhibitors**	-CTLA-4 inhibitors: ipilimumab -PD-1 inhibitors: nivolumab, pembrolizumab -PD1l inhibitors: atezolizumab	Exanthema (rash)	-Mild (<30% BSA): oral antihistamines, topical steroids	IV
			-Moderate (>30% BSA): oral steroids (1–2 mg/kg); hold immunotherapy	IV
			-Severe (SSJ/TEN or necrotic, bullous or hemorrhagic complications): systemic steroid (1–2 mg/kg); permanently discontinue immunotherapy	IV
		Pruritus	*Prevention:*	
			Limited shower time, use of gentle cleansers (pH-neutral soaps or syndets), regular use of emollients	IV
			*Treatment:*	
			-Oral antihistamines	IV
			-Topical or oral steroids	IV
			-GABA agonists (pregabalin, gabapentin)	IV
		Oral toxicity -Lichenoid lesions -Dry mouth	Topical steroids and anesthetics, good oral hygiene	IV
		Vitiligo	No definitive treatment	IV
		Others:	Only case reports on the literature	
		-Lichenoid eruptions		
		-Sarcoidosis		
		-Auto-immune blistering diseases (bullous pemphigoid)		
		-Psoriasis		

BSA, Body Surface Area; CTLA-4, Cytotoxic T Lymphocyte Associated Antigen 4; PD-1, Programmed Death 1; PD-1l, Programmed Death 1 Ligand; SSJ, Steven-Johnson Syndrome; TEN, Toxic Epidermal Necrolysis.

* Dual checkpoint blockade (anti-CTLA-4 + anti-PD1) is related to a higher grade of adverse events, including dermatologic toxicities. Case reports for severe irAE are available with the use of other immunomodulatory agents.

Categories of evidence based on types of studies^3^:

IA – Evidence from meta-analysis of Randomized Controlled Trials (RCT);

IB – Evidence from at least one Randomized Controlled Trial (RCT);

IIA – Evidence from at least one controlled study without randomization;

IIB – Evidence from at least one other type of experimental study;

III – Evidence from non-experimental descriptive studies, such as comparative studies, correlation studies and case-control studies;

IV – Evidence from expert committee reports, opinions or clinical experience of respected authorities, or both.

## Categories of agents

### Conventional chemotherapeutic drugs

Conventional cytotoxic chemotherapy still plays an important role in cancer treatment. It works primarily through the inhibition of cell division. It is associated with many adverse events (AE), especially in some systems that share with the tumor the property of rapid cell proliferation and therefore a high rate of cell division, such as the hematopoietic and gastrointestinal systems, and the skin.^2^, ^4^ Examples of common effects are emesis, cytopenias, alopecia, mucositis and nail changes. They are associated with dose, type of drug, time of infusion and are in most cases reversible with the end of chemotherapy cycles.

Belonging to this class of drugs are agents such as antimetabolites (e.g. capecitabine, fludarabine, cladribine, gemcitabine, 5-fluoracil), alkylating agents (e.g. cyclophosphamide, platins), topoisomerase inhibitors (e.g. irinotecan, topotecan, etoposide), anthracyclines (e.g. doxorubicin, daunorubicin), bleomycin, antimicrotubule agents (e.g. taxanes and vinca alkaloids). The related cutaneous adverse events to this class, most frequent causing agents and management (with level of evidence) are summarized in table 1.

### Targeted therapies and immunomodulatory agents (“checkpoint” inhibitors)

In the last decade an enormous development on oncologic treatments have occurred, with the emergence of numerous targeted agents and immune checkpoint related agents. With those new mechanisms of action a totally new scope of adverse reactions has arisen, and professionals may not be familiar with the spectrum of dermatological toxicities.^5^, ^6^, ^7^, ^8^ In one hand, those therapies were crucial for the improvement of survival. On the other hand, they created a new challenge: with the continuous and prolonged use of these drugs, patients and professionals have to deal with the chronic aspects of the toxicities, not anymore related with a specific number of chemotherapy cycles, but lasting for months to years, with an important impact on quality of life.

Targeted therapies inhibit specific molecules involved in tumor development and growth, having a more specific action than conventional chemotherapy, with greater efficacy and less toxicity. Many of those molecules are mutated or overexpressed on tumors, but are also present in normal tissues such as the skin. This justifies the common dermatological side effects related to this class. They might be monoclonal antibodies, large molecules that target extracellular components; or small molecule inhibitors, that can enter cells, block receptor signaling, and interfere with downstream intracellular molecules (they will be referred as Tyrosine Kinase Inhibitors – TKI).^9^ The main agents on this class are Epidermal Growth Factor Receptor Inhibitors – EGFRi (e.g. cetuximab, panitumumab, gefitinib, erlotinib, lapatinib); antiangiogenic agents, inhibitors of Vascular Endothelial Growth Factor Receptor – VEGFRi (e.g. bevacizumab, sorafenib); inhibitors of the Mitogen-Activated Protein Kinase (MAPK) pathway, such as RAFi (e.g. vemurafenib, dabrafenib), MEKi (e.g. cobimetinib, trametinib); mTOR inhibitors (e.g. everolimus); multikinase inhibitors (e.g. vandetanib, pazopanib, sunitinib); and Hedgehog pathway inhibitors (vismodegib). Related cutaneous adverse events to the agents on this class, their management and levels of evidence are summarized in table 2.

More recently, the comprehension of the regulatory processes involved in the restriction of the immune response to cancer, led to the development of a new promising group of agents, the “immune checkpoint” targeted agents, also known as immunotherapy. They have the goal of releasing the immune system against tumor cells, blocking inhibitory receptors expressed on T-cells such as Programmed Death 1 (PD-1) or Cytotoxic T Lymphocyte-associated Antigen 4 (CTLA-4). This releasing/activation of the immune system also affects normal cells, explaining the most common adverse events related to this class, also known as Immune-Related Adverse Events (irAE). On this group we find the CTLA-4 inhibitor (ipilimumab), PD-1 and PD-1 ligand inhibitors (nivolumab, pembrolizumab, atezolizumab).^7^, ^10^, ^11^, ^12^, ^13^ Related cutaneous adverse events to the agents on this class, their management and levels of evidence are summarized on table 3.

## Grading the dermatological adverse events

The National Cancer Institute's Common Terminology Criteria for Adverse Events (CTCAE) is a standardized tool used in oncology trials to document and grade toxic effects of oncologic therapies. It is available online with open access at https://ctep.cancer.gov/protocolDevelopment/electronic_applications/ctc.htm.

It divides AE into systems and has a specific chapter for skin and subcutaneous tissue disorders. It helps establish a common terminology and language between different experts (oncologists, dermatologists, nurses, etc.) and to standardize treatments or dose modifications based on the severity of specific manifestations.

## Impact of dermatological adverse events on quality of life

Quality of life is an important issue when dealing with oncologic patients. Different studies have already shown that grading severity of AE are different from the physician and the patient's perspectives.^14^ Therefore, using patient's self-reporting instruments for DAE are also important. Different instruments are available such as Skindex-16©, Skindex-29©, Dermatology Life Quality Index (DLQI), DIELH-24 or even questionnaires for specific agents such as the Functional Assessment of Cancer Therapy-Epidermal Growth Factor Receptor Inhibitors-18 (FACT-EGFRI-18).^15^

When comparing different classes of drugs, the number of DAEs and its impact on QoL seemed to be greater on patients on targeted versus non-targeted therapies (difference = 8.9, *p* = 0.02).^15^

## Cutaneous toxicity as a predictive marker for clinical outcome

A recent review tried to address the association of cutaneous toxicities and clinical outcome. For papulopustular eruption induced by EGFRi, this association has already been established (four trials, all with *p* < 0.05). For hand-foot skin reaction associated with sorafenib, it was associated with reduced risk of death on a systematic review of 12 cohort studies (*p* < 0.00001; hazard ratio = 0.45). However, authors concluded that for other toxicities such as vitiligo and immunotherapy for melanoma (data based mostly on retrospective analyses), the analyses are still observational and exploratory and need further investigation from larger prospective studies.^16^

## Daily baseline skin care

Xerosis is a common dermatological condition in patients on cancer treatment, with incidences ranging from 1 to 84% and can collaborate to a decrease in QoL and to the occurrence of other DAE, such as infections, sensitization to allergens and pruritus. It is even more frequent with the use of targeted therapies (for xerosis – RR = 2.99, 95% IC 2.0–4.3, *p* < 0.001; for pruritus RR = 2.56, 95% IC 1.51–4.35, *p* < 0.001).^17^, ^18^ The skin also becomes more sensitive to the ultraviolet radiation and more prone to skin pigmentation. Therefore, it is fundamental to educate patients on preventive measures, even prior to the start of any therapy, that help maintaining skin barrier function, possibly decreasing the occurrence and severity of DAE.

Some important measures to all cancer patients are: avoidance of alcohol- based lotions and irritating products, limited shower time, use of gentle cleansers (pH-neutral soaps or syndets), regular use of emollients and sun protective measures (e.g.: broad spectrum sunscreen – SPF30 or higher with a UVA-PF, protective clothes).^1^

The use of deodorants is controversial, but some data show no evidence of harm, and therefore, patients might use those products in order to maintain their regular routine and help on their well-being.^19^ The use of make-up or camouflage may also help on the self-esteem. Preference for noncomedogenic products is recommended.

## Dermatological adverse events

As dermatologists usually start their evaluation on skin exam, the different DAE will be grouped in types of skin reactions and areas of involvement. In Table 1, Table 2, Table 3, readers can find the DAE grouped by class of agents and the level of evidence of the recommendations for each treatment used on this guideline.

### Pigmentary changes

**Figure 1:**
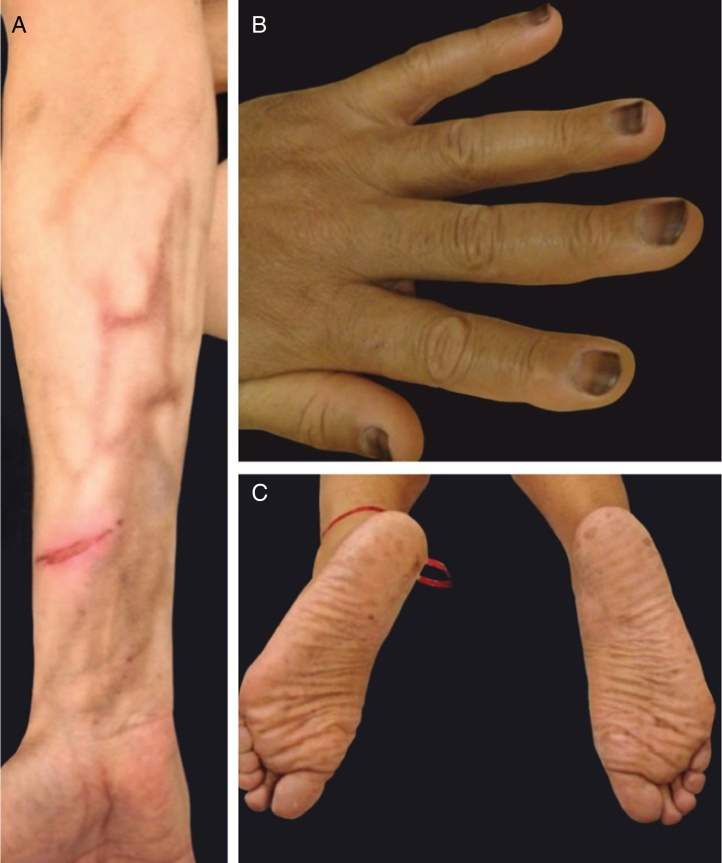
Different hyperpigmentation patterns: (A) serpentine supravenous hyperpigmentation after peripheral chemotherapy infusion (fluorouracil); (B) nail plate pigmentation (daunorubicin); (C) acral lentiginoses (doxorubicin).

Hyperpigmentation can occur at different sites and in different patterns (Fig. 1). They can start days to months after initiation and most of the times fade months after discontinuation of therapy. It can occur: (a) On photo distributed areas, preceded or not by photosensitivity signs; (b) As a serpentine supravenous hyperpigmentation after peripheral chemotherapy infusion (e.g. fluorouracil, docetaxel), that might be preceded or not by erythema and inflammation; (c) In a diffuse pattern, sometimes reticulated (doxorubicin, hydroxyurea, methotrexate); (d) Localized in areas of pressure, flexural areas or under occlusive dressings (ifosfamide, thiotepa); (e) As acral pigmentation (along the crease lines or macular, over the palms and soles).^4^, ^20^

**Figure 2:**
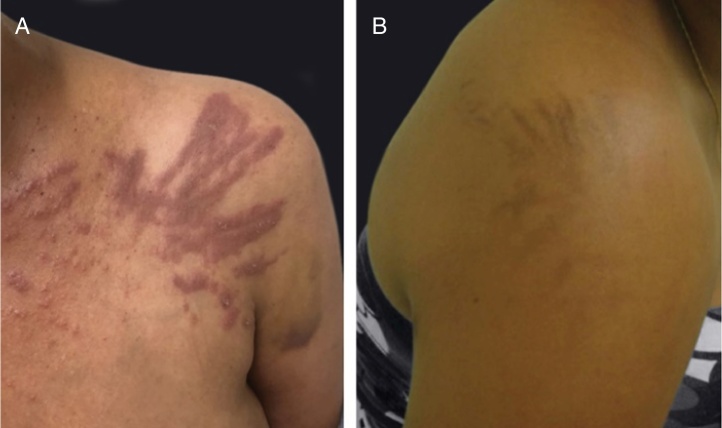
Flagellate dermatitis associated to bleomycin treatment: (A) pruritic erythematous linear streaks, (B) followed by linear pigmentation.

Flagellate dermatitis and pigmentation is related to bleomycin treatment (20%–30% of patients) and occur as a pruritic erythematous linear streaks, followed by pigmentation (Fig. 2). Busulfan treatment can cause an “Addison-like” pigmentation, with a tan appearance.^4^

Patients should be oriented to adhere to sun-protective measures and bleaching agents might also be used to accelerate clearing.

Targeted therapies can also cause pigmentary changes such as a yellow pigmentation on sunitinib treated patients (VEGFRi). Hypopigmentation (diffuse or localized) is associated with c-kit inhibitors such as imatinib, especially in patients with darker skin, as c-kit also regulates melanocyte function. They are usually reversible with dose reduction or discontinuation.^5^

More recently, immunotherapy has been linked to vitiligo-like lesions, mainly but not only in melanoma treated patients. Associated hair depigmentation can also be observed. No definitive treatment exists and it usually persists after the completion of immunotherapy. Reports of white hair repigmentation are also available.^10^, ^11^, ^12^, ^21^

### Nail changes

Nail alterations during cancer treatment are frequent, usually well tolerated and disappear under cessation of treatment. However, in some circumstances they might be symptomatic and interfere with patient's daily activities.^22^

Conventional chemotherapeutic agents are usually related to alterations such as melanonychia (diffuse, transverse or longitudinal), leukonychia, onycholysis, Beau's lines, onychomadesis and onychorrhexis.

**Figure 3:**
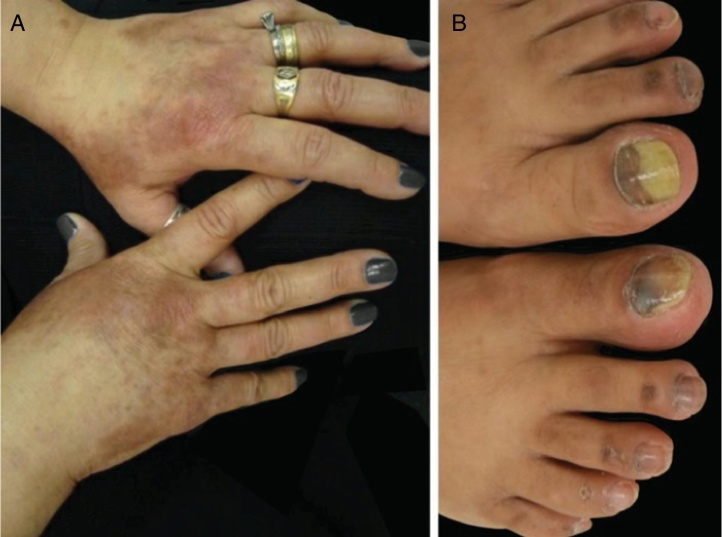
PATEO syndrome (PeriArticular Thenar Erythema and Onycholysis): docetaxel treated patient presenting with (A) erythematous lesions with a distinct distribution to the dorsal aspects of the hands and (B) associated nail changes – subungual hemorrhage and onycholysis.

Taxanes are associated with painful subungual hemorrhage, followed by onycholysis and sometimes subungual abscess formation that are quite symptomatic, and might lead to dose reductions or treatment discontinuation (Fig. 3). The use of frozen gloves and socks during infusion might reduce the severity of nail alterations, but they are usually not well tolerated (nail toxicity, one study, 45 patients, side-to-side, 11% vs. 51%, *p* = 0.0001).^2^, ^4^, ^20^ Drainage of hemorrhage and abscesses might be needed for symptoms relief, as well as culture guided antibiotic treatments.

Both conventional and targeted treatments can cause nail plate alterations such as brittle nails, nail cracking and onychoschizia. Those effects can be handled with preventive measures such as reduction of contact to water, use of cotton gloves beneath plastic or rubber gloves for any wet work, avoidance of any damaging procedure (e.g.: aggressive manicuring), and hydration with thick emollients. Oral biotin and lacquers such as hydroxypropyl chitosan and poly-ureaurethane might be used, although no controlled study is yet available.^22^

For onycholysis, avoiding traumas (e.g.: keeping nails short) and humidity is important. If signs of pseudomonas colonization (green discoloration), a topical antibiotic might be used (we suggest ophthalmologic solutions such as tobramycin or ciprofloxacin eye drops twice daily).

VEGFRi are associated with asymptomatic splinter subungual hemorrhages (black, red or brown longitudinal lines) in 25–70% of patients, with fingernails being mostly affected.^5^, ^7^

**Figure 4:**
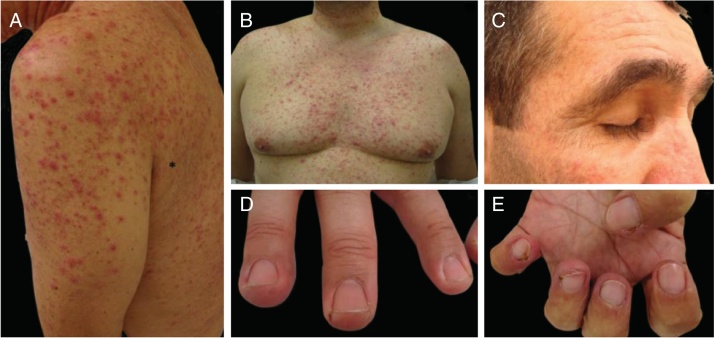
EGFR inhibitors related adverse events: (A and B) inflammatory papulopustular rash with associated xerosis (*); (C) trychomegaly and hypertrichosis; (D) periungual fissures and (E) pyogenic granuloma-like lesions. (A, B, D and E on cetuximab treated patients; C on panitumumab treated patient).

EGFRi are related to frequent nail toxicities, occurring in around 17.2% of patients on a previous meta-analysis.^23^ Periungueal fissures, paronychia and pyogenic granuloma-like lesions start to develop two or more months after initiation of therapy, on both finger and toe nails (Fig. 4). They are initially sterile, but secondary infection may occur. Preventive measures include the ones listed above and also the use of comfortable shoes, sometimes with cushioning inserts for the affected nails, adequate nail cutting and the use of antiseptic solutions such as Burrow's solution soaks, white vinegar soaks (1:1), bleach soaks (1/4 cup bleach: approx. 10 l water), chlorhexidine or povidone iodine^6^, ^24^, ^25^, ^26^ or a solution of sodium hypoclorite 2.5%, sodium chloride 1.0%, deionized water qsp 100 mL (5 drops/1 l). The use of oral tetracyclines is controversial as they were related to a decrease on the incidence of paronychia in some trials and showed no benefits on others.^27^ For fissuring, protective coverings such as hydrocolloid, biological glue or even cyanoacrylate glue to relieve pain and promote healing, barrier creams (petroleum jelly, zinc oxide cream) and thick emollients might be used. For granulomas, if secondary infection is suspected, culture is indicated and proper antibiotics should be prescribed. For non infected lesions, destructive measures such as trichloroacetic acid (70–90%) or cryotherapy might be used. Potent topical steroids, occlusive or not, are also indicated.^28^ Sometimes surgical treatment might be necessary. Recently, the use of topical betaxolol was described for treating a pyogenic granuloma-like lesion induced by EGFRi.^29^

### Acral reactions

**Figure 5:**
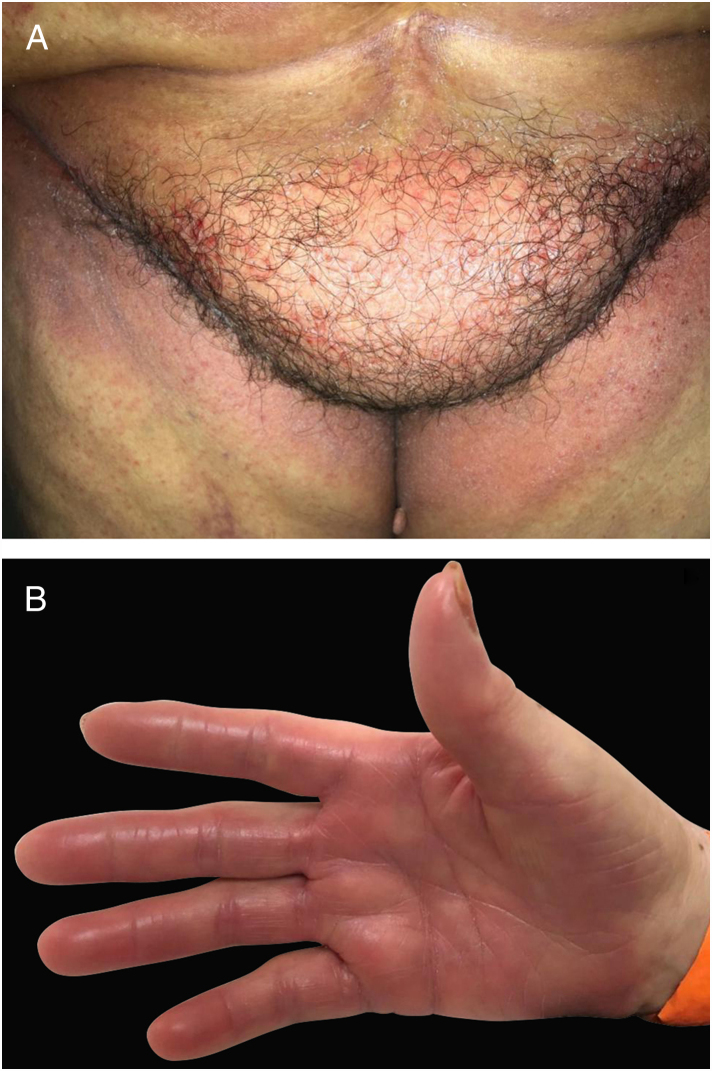
Toxic erythema of chemotherapy (TEC): combination of different lesions caused by direct toxicity of chemotherapy agents with (A) lesions on flexural areas (intertriginous eruption associated with chemotherapy) and (B) on palms and soles (Hand-foot syndrome – HFS).

Hand-foot syndrome (HFS), also known as palmoplantar erythrodysestesia, is a DAE related to conventional chemotherapeutic drugs, common with agents such as capecitabine, doxorubicin, cytarabine, 5-FU. It is preceded by prodrome symptoms such as tingling or pain at the extremities, followed by a symmetric, sharply demarcated erythema and edema of the palms and soles (Fig. 5). Vesicles and bullae might also be present. Pain usually limits daily activities and might be a cause of dose reduction or discontinuation. Incidence is higher with prolonged infusions or oral agents.^2^, ^4^

PATEO syndrome (PeriArticular Thenar Erythema and Onycholysis) is a variant of HFS, specific to the taxanes (docetaxel) (Fig. 3). It is characterized by scaly erythematous lesions with a distinct distribution to the dorsal aspects of the hands (overlying the joints) and thenar eminences. More rarely, it can also affect the dorsum of the feet. Although common, nails are not universally affected. It is usually bilateral, not necessarily symmetric. It may start on the first chemotherapy cycle or develop progressively. Burning sensation and pain are the most reported symptoms.^20^

**Figure 6:**
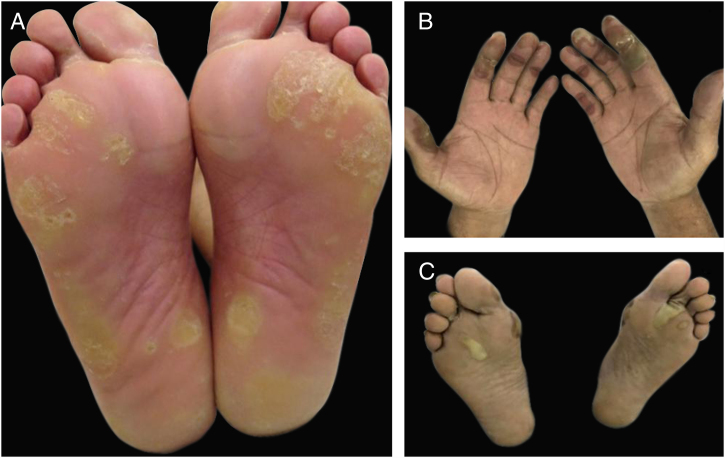
Hand-foot skin reaction (HFSR) associated with antiangiogenic agents (VEGFRi): (A) hyperkeratotic lesions (sorafenib) and (B) bullous lesions (axitinib) on areas of pressure and friction.

Hand-foot skin reaction (HFSR) is another variant of acral toxicity related to targeted therapies, with both monoclonal antibodies and small molecules tyrosine kinase inhibitors especially with those targeting VEGFR (bevacizumab, sorafenib, sunitinib) and BRAF (vemurafenib) (Fig. 6). Despite the similar palmoplantar distribution, dose dependency and associated pain, HFSR develop on friction or trauma-prone areas (pressure areas), such as the heel and lateral aspects of the soles and web spaces. Lesions start 2–4 weeks after beginning of therapy and are characterized by hyperkeratosis, resembling skin calluses, occasionally with superficial blistering and erythematous halos.^2^, ^5^, ^6^, ^30^, ^31^ Depending on the drug, lesions might improve or not over time.^32^

#### Prevention

HFS and PATEO: on a prior systematic review, the only two evidenced based measures for prevention (to decrease incidence and/or severity) were the use of a nonsteroidal anti-inflammatory drug (NSAIDs – celocoxib) (for any grade: Odds Ratio – OR = 0.47, 95% IC 0.29–0.78, *p* = 0.003; for Grade 2–3: OR = 0.39, 95% IC 0.20–0.73, *p* = 0.003) and dose reduction. One study showed benefit with the use of regional cooling during chemotherapy infusion (incidence 36% × 7.1%, *p* = 0.0097). However, as previously discussed, frozen gloves and socks are usually not well tolerated by patients; regarding to NSAIDs, the risks and benefits of their use must be weighted; regarding dose reductions, it has direct impact on disease outcome.^33^, ^34^ The use of emollients seemed promisor, but with no statistical significant difference. Pyridoxine was not effective for the prevention.^33^, ^34^, ^35^ A small RCT showed benefits with the use of an antioxidant-containing ointment when compared to placebo on pegylated liposomal doxorubicin treated patients.^36^ For HFSR one RCT showed a decreased incidence in any grade and >Grade 2 reaction with the use of urea 10% cream compared to “best supportive care” (for any grade: OR = 0.457, 95% CI 0.34–0.60, *p* < 0.001, for ≥Grade 2: OR = 0.635, 95% CI 0.46–0.86, *p* = 0.004). Results might be questioned, though, because “best supportive care” was indeed no preventive care.^37^, ^38^ Another small prospective trial showed a decrease in occurrence of HFSR on sorafenib treated patients with the ingestion of a Japanese food (Bonito broth) when compared to no ingestion (Hazard Ratio, HR = 0.097, 95% CI 0.011–0.846, *p* = 0.035 on multivariable analysis). This food is shown to increase peripheral blood flow in humans.^39^ Another isolated report showed success with the use of topical calcipotriol in one case.^40^

Despite the absence of a strong evidence, we do recommend the following preventive measures^2^, ^5^, ^6^, ^7^, ^30^, ^31^, ^41^:

Educate patients of early signs and symptoms;

Use thick cotton gloves and/or socks;

Apply emollient creams (urea based emollients in hyperkeratotic type) to hands and feet regularly;

Avoid irritants such as alcohol, harsh cleansing agents and tight clothing and shoes;

Avoid extremes of temperature, pressure and friction (e.g.: repetitive activities, stressful manual work, etc.).

For HFSR also include a pretreatment evaluation with a podiatrist with callosity chopping and the use of orthopedic shoe inserts when needed.

#### Treatment

Also low evidence exists on treatment measures. Dose reductions are effective but interferes with disease related outcomes. One small prospective non-comparative trial showed improvement in QoL and decrease on HFS symptoms with the use of a topical non-occlusive polymer for 8 weeks.^42^ For HFSR, one small randomized phase II trial showed benefit with the treatment of Grade 1 toxicity with a hydrocolloid dressing containing ceramide with a low-friction external surface when compared to urea 10% cream (Grade 2–3, 29% vs. 69%, *p* = 0.03).^43^ Recently, a systematic review showed benefits of different Chinese herbs on the treatment of acral toxicities. However, most of the studies were not blinded and with a lower quality.^44^, ^45^ One case report and a small case series suggest benefits on the use of topical Henna for capecitabine HFS.^46^, ^47^ Besides the already mentioned preventive measures, recommendations of this board for the treatment of those toxicities include^2^, ^5^, ^6^, ^7^, ^30^, ^31^, ^41^:

Continuing the use of preventive measures;

Maintaining the use of emollients, and on the case of HFSR, include the use of keratolytic agents (e.g.: urea 10–40%, salicylic acid, etc.);

Add a potent topical steroid;

For relief of symptoms, cool compresses or emergence of hands and feet on cool water, topical anesthetics and NSAIDs might be used;

Dose reduction or treatment interruption is sometimes necessary until symptoms decrease.

### Skin rashes

One of the problems of better defining the DAE in many oncology trials is that frequently investigators report the different types of cutaneous eruptions as a skin “rash”. Yet, as we will see, there are different types of cutaneous eruptions, associated with different classes of drugs and with different treatment options.

Acute hypersensitivity reactions: those Type I immunoglobulin E-mediated reactions may occur within minutes to hours of infusion. They manifest as regular hypersensitivity reactions to conventional drugs, as pruritus, flushing, urticaria and even anaphylaxis. The difference relies though on the way we deal with it. With conventional drugs, patients are usually oriented to avoid re-exposure. When dealing with oncologic treatments, the chemotherapeutic agent is fundamental for disease related survival. Therefore, we usually maintain the drug for the next cycles and manage the reaction with a slower infusion, a premedication with corticosteroids and antihistamines before every infusion and a closer monitoring.^4^, ^6^, ^7^

Exanthema: many treatments may be related to a non-specific maculopapular rash or morbiliform eruption that starts gradually, sometimes weeks after the start of drug, with mild symptoms such as pruritus. Those DAE can be handled with anti-histamines and topical corticosteroids when limited, or with short courses of oral corticosteroids when more disseminated. All class of agents might cause those kind of reactions, such as kinase inhibitors (e.g.: BRAFi – vemurafenib/kit and BCR-ABL inhibitors – imatinib, dasatinib), “checkpoint” inhibitors (ipilimumab, nivolumab), and conventional chemotherapeutic agents (bleomycin, carboplatin, etoposide, etc.).^4^, ^6^, ^7^ Only in rare occasions severe reactions such as toxic epidermal necrolysis (TEN), Steven-Johnson syndrome (SSJ) or drug rash with eosinophilia and systemic symptoms (DRESS) might occur, but it must be remembered that a maculopapular rash may represent the first manifestation of those life-threatening conditions. Special attention should be given when using a targeted therapy after the use of an immunotherapy (e.g.: melanoma patients treated with immunotherapy and then switched to a BRAFi) as it may be associated with a higher risk of severe skin toxicity (> Grade 3 and SSJ/TEN). Some authors suggest at least a 4 week interval between treatments with those agents.^7^, ^48^, ^49^, ^50^

When a patient is on immunotherapy, skin adverse events to conventional drugs (e.g.: antibiotics) might be more intense, so it is always important to exclude other causative agents, before relating the rash to the “checkpoint” inhibitor.

Toxic erythema of chemotherapy (TEC): this term is suggested by some authors to unify different manifestations such as HFS, intertriginous eruption of chemotherapy or other used histopathologic terms such as “eccrine squamous syringometaplasia”, all related to a direct toxicity of the chemotherapeutic agent and not due to an allergic reaction. This type of DAE is characterized by overlapping features of bilateral painful erythema, edema, and even bullous lesions located on hands and feet (see hand-foot syndrome), and sometimes also affecting intertriginous areas such as axilla and groins (less frequently ears, knees and elbows) (Fig. 5). It is important to distinguish, because those manifestations are usually self-limited, often resolving with desquamation and post-inflammatory pigmentation and not demanding aggressive measures. In addition, lesions develop 2–3 weeks after the chemotherapy cycle, usually when patient's defenses are lower (e.g.: neutropenia), being many times misdiagnosed as infections or graft-versus-host disease (GVHD). Treatment relies on topical corticosteroids and emollients, and educating the patient about the nature of the manifestation. It often recrudesces on the subsequent cycles. It might be milder with dose reductions.^20^, ^51^

Papulopustular rash: also known as “acne-like rash” or “folliculitis”, this is the most common dermatological toxicity of EGFRi treatment (Fig. 4). It usually appears 1–2 weeks after initiation of therapy, starts with erythema, followed by the eruption of papules and pustules (sterile) on the face, scalp, upper chest and back, with a lack of comedones. Sometimes lesions extend to the limbs. Skin is usually dry, itchy and sensible (patients refer sensation of burning, stinging, tenderness). This AE has a high impact on patient's QoL and on social aspects of daily living, being a cause of dose reduction or even treatment discontinuation. The rash tends to improve around the 8th week, but usually persists, as a milder eruption, with periods of improvement and worsening. Other features that are present, especially later on treatment, are the already discussed paronychia, periungual fissures and granulomas, xerosis, pruritus and the yet to be discussed hair alterations such as trichomegaly, hypertrichosis and non-scarring alopecia.^6^, ^23^, ^24^, ^25^, ^52^

#### Prevention

General daily baseline measures have already been discussed and are of great importance on this group of agents. They include the regular use of emollients, photo protective measures (sun exposure might worsen the eruption), avoidance of irritating agents, limited shower time and use of gentle cleansers. The use of systemic antibiotics (mostly tetracycline agents) on the first 6–8 weeks of treatment has been evaluated in some trials with discordant results. The available data suggest some benefit of the preventive treatment in decreasing the incidence and mainly the intensity of the papulopustular rash when compared to no treatment or even to the reactive treatment (started once the rash is already present).^26^, ^53^, ^54^ This is also the opinion of this expert panel. We suggest the use of oral tetracyclines (e.g. doxycycline 100 mg bid.) during the first 6 to 8 weeks of treatment with EGFRi. On one randomized phase II trial, topical erythromycin was inferior to oral doxycycline for the prevention of EGFR skin toxicity.^54^ Another trial showed some benefit with the preventive use of low potency topical steroids, but we recommend their use only as a reactive treatment.

#### Treatment

Despite the acne-like appearance, topical agents used to treat acne and acneiform eruptions such as benzoyl peroxide, retinoic acids and salicylic acid containing products are contra-indicated. If the rash occurs, preventive measures should be maintained and to that, topical steroids can be added. If more pronounced and tetracycline was not started for prevention, it can now be initiated.^6^, ^23^, ^24^ Sometimes dose reductions might be necessary. If excessive crusting or secretion, cultures are indicated to exclude secondary bacterial infection. In those cases, culture guided antimicrobial treatment is indicated. Recently, a single RCT showed benefit with the use of a Chinese herbal topical compound (compared to placebo) for the treatment of the DAE related to targeted therapy, including the papulopustular rash.^45^ In addition, there are some reports and some personal experience on the use of low dose systemic isotretinoin for refractory cases.^55^

Acneiform eruption: conventional chemotherapeutic regimens frequently contain high doses of systemic corticosteroids, such as dexamethasone or prednisone. For that reason, steroid related acneiform eruption might occur and should be treated similar to the non-oncologic patients.

Photosensitivity reactions: oncologic treatments have been reported to cause both phototoxic or photoallergic reactions. Many conventional agents such as 5-FU and taxanes (mainly to UVB) have reported inflammatory rashes on photo-exposed areas.^4^, ^20^ With EGFRi, not only photosensitive eruptions can occur, but also the other DAE can be exacerbated by photo exposure.^6^ One of the most photosensitizing agent is vemurafenib (BRAFi) with a proved sensitivity for UVA radiation, which is present on fluorescence lamps and passes through windows.^5^, ^7^, ^56^, ^57^ Vandetanib (TKI) is also associated to a UVA sensitivity. Preventive sun protective measures are fundamental. If reaction occurs, it might be handled with topical steroids or short courses of oral steroids.

**Figure 7:**
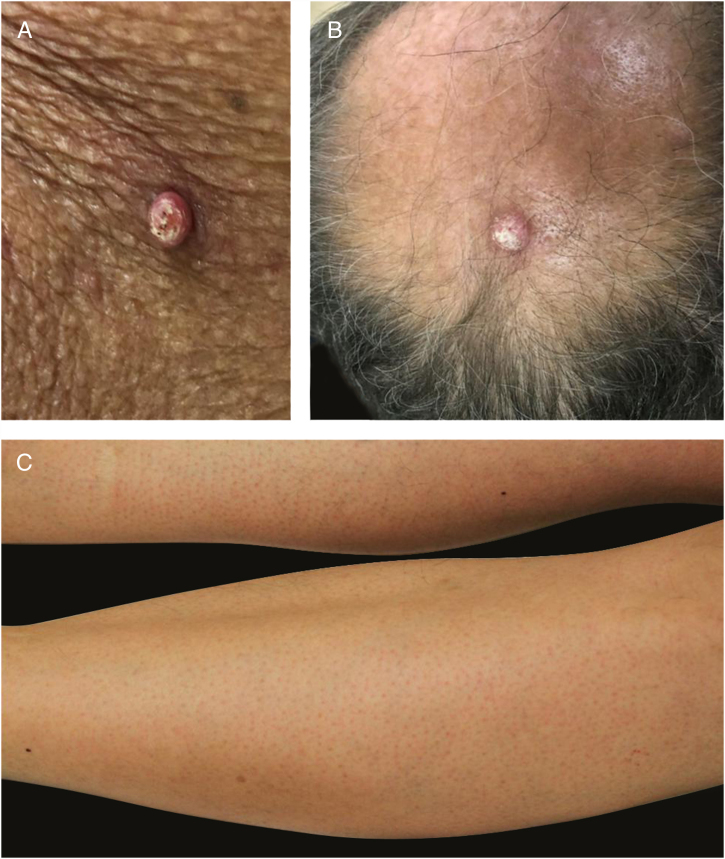
BRAF inhibitor related adverse events: multiple keratoachantomas (A) and low grade squamous cell carcinomas (B) after withdrawal of MEK inhibitor and maintenance of BRAF inhibitor; (C) associated keratosis pilaris-like eruption on the lower limbs.

Keratosis pilaris-like eruption: this adverse event has been linked to the use of BRAFi and is characterized by diffuse follicular keratotic papules in a generalized distribution, resembling keratosis pilaris. Topical keratolytic agents might be used.^7^ Vemurafenib could also cause folliculocentric morbilliform rash (Fig. 7).

### Scalp and hair abnormalities

Some general recommended measures for hair and scalp daily care include the use of a gentle shampoo, avoiding hot water, hair dyes and hair foaming.

Conventional chemotherapy-induced alopecia (CIA): is one of the most distressing events in cancer patients treated with conventional agents. It is caused mainly by an anagen effluvium and is usually (although not always) completely reversible 2–6 months after treatment is discontinued.^2^, ^20^ It affects more often scalp hair, but eyebrows, eyelashes, and other body areas might also be affected. Hair loss will be influenced not only by the drug, but also by the route of administration, dosing and schedule (e.g.: high dose, intravenous, intermittent regimens are more prone to cause complete alopecia). Examples of agents with a high risk include cyclophosphamide, doxorubicin, irinotecan and taxanes (docetaxel and paclitaxel).

#### Prevention

A systematic review of preventive measures found no benefit with the use of topical minoxidil, or scalp compression. Benefit was found with the use of a scalp cooling device (RR = 0.38, CI 95% 0.32–0.45, *p* < 0.001).^58^, ^59^, ^60^, ^61^ Scalp cooling systems include static devices (cool caps) and dynamic scalp cooling systems. Patients should be warned that treatment efficacy is variable (around 50–80% depending on agent). Available data suggest that this technology is most effective for taxane-based chemotherapy regimens compared with anthracycline-based chemotherapy regimens.^62^ Besides, the use of the cooling devices are not always well tolerated as they may cause symptoms like headache and scalp pain. Devices are also not reimbursable by insurance companies and might have a high cost. Recent studies have shown that the incidence of scalp metastasis is not increased in breast cancer patients with localized disease treated with scalp cooling.^63^, ^64^ However, there are two reported cases of disease recurrence in patients with hematological malignancies (mycosis fungoides and acute myeloid leukemia).^65^, ^66^ Because of that, scalp cooling should be avoided in hematological malignancies.^67^

#### Treatment

Minoxidil was not effective in preventing CIA, but a small trial showed that minoxidil 2% was associated with a faster regrowth of hair (time to regrowth of 86 vs. 136 days on the placebo group).^68^ Other agents such as topical calcitriol are under investigation, but still with no definitive results.^2^, ^69^ Based on the studies and on authors daily practice, we recommend the use of topical minoxidil 5% once daily after the end of the chemotherapy cycles. For eyebrows and eyelashes, topical bimatoprost might be used (one controlled study for eyelash showed benefit at 12 months).^70^ Biotin and other oral supplements might be added. Camouflage and support for patients are also important strategies.

Reversibility: CIA is completely reversible in most of the cases. When incomplete/suboptimal hair regrowth occurs after 6 months of discontinuing therapy, it is considered a persistent CIA (pCIA). It has a usually diffuse, non-scaring pattern and occur more often with pre bone marrow transplantation high dose regimens (usually busulfan and cyclophosphamide) or with taxanes.^20^, ^71^

Another frequent pattern of persistent alopecia on cancer patients is described as endocrine therapy-induced alopecia. For many hormone receptor-positive breast cancer survivor's selective estrogen receptor modulators (e.g., tamoxifen, toremifene), aromatase inhibitors (e.g., anastrozole, letrozole, exemestane) and gonadotropin-releasing hormone agonist (e.g., leuprolide) are usually administered for 5–10 years to reduce the risk of recurrence.^5^, ^6^, ^7^, ^8^, ^9^, ^10^ This estrogen deprivation might lead to androgenetic pattern alopecia. Topical minoxidil can be used. As for the use of systemic therapies for androgenic alopecia (e.g., spironolactone, finasteride), there is a putative risk of hormonal stimulation of endocrine receptor-positive tumors, so the use of these agents must be discussed with the oncologist and used with caution.^67^, ^71^

Targeted therapies induced hair abnormalities: changes in hair quality, texture and growth pattern might be seen around the 2nd or 3rd month of treatment.

Scalp: scalp hair grows slower and with a fragile quality. A seborrheic dermatitis-like rash may develop (especially with VEGFRi and BRAFi). Also the papulopustular rash affecting the face and trunk might involve the scalp (especially with EGFRi). Alopecia is usually mild and with an androgenetic pattern, but cases of inflammatory non-scarring alopecia have been described, as cases of erlotinib-induced cicatricial alopecia.^5^, ^6^

For those inflammatory changes, we suggest the use of anti-dandruff shampoos and topical corticosteroids (lotion or shampoo). If bacterial infection is suspected, cultured guided antibiotics are indicated.

Face, eyelashes and eyebrows: trichomegaly (longer, thicker and often curled eyelashes) and hypertrichosis are frequent (Fig. 4). Inward eyelashes may result in keratitis, therefore eyelash clipping is advised and patients with ocular symptoms should be referred to an ophthalmologist. For hypertrichosis, topical, cosmetic interventions can be used (e.g. waxing or bleaching). When available laser and photoepilation treatments are most effective and are not contra-indicated.^69^ Creams for epilation should be avoided due to their sensitizing potential in those subjects that already have a skin barrier dysfunction.

Immunotherapy induced hair abnormalities: cases of alopecia with a clinical and histologic pattern consistent with alopecia areata (AA) have been reported. Patients should be treated similarly to non-oncologic AA cases. Also reports of hair depigmentation and repigmentation have been described. Even though we advise patients to avoid hair dyeing, it is not contra-indicated, and if there is an important cosmetic concern, dyes might be used.^69^

### Changes in melanocytic nevi

BRAF inhibitors: might be associated to the appearance of eruptive melanocytic nevi (EMN), change of preexisting nevi (both the increase and acquisition of dermatoscopic structures as well as the regression of nevi that have the BRAF mutation) and the appearance of new melanomas.^72^, ^73^ Therefore, close dermatoscopic follow-up is recommended. The involution of BRAF inhibitor-induced EMN following the concomitant addition of MEK inhibitor has been described.^74^

Immunotherapy: recently, regression of multiple melanocytic nevi after immunotherapy for melanoma has been described.^75^, ^76^

### Other particular toxicities

Epidermal neoplasms related to BRAF inhibitors: keratoacanthomas, squamous cell carcinomas and verrucal keratosis are a common DAE of BRAFi, they might also occur with some antiangiogenic agents (Fig. 7). This is probably due to the paradoxical stimulation of the MAPK pathway in BRAF wild-type cells. When MEK inhibitors are used in association, this AE is much less frequent. Lesions can be treated with surgical excision (when only a few lesions are present), destructive treatments (cryotherapy, curettage, etc.), topical treatments (keratolytics, 5-FU, imiquimod) or photodynamic therapy.^7^

Stomatitis related to mTOR inhibitors (rapamycin, everolimus, sirolimus): stomatitis is the most common AE and might be severe leading to dose adjustments. Different from conventional chemotherapy mucositis (broad ulceration with pseudo membrane formations), mTOR related stomatitis manifests as discrete aphthae on non-keratinized epithelium. They can be handled with antiseptic washes, topical steroids and anesthetics.^7^

Other eruptions related to immunotherapy: a non-specific maculopapular rash, as reported above, represents the most prevalent type of Immune-Related Adverse Events (irAE) to this class of agents. However, peculiar types of reactions have been described and include:

Lichenoid reactions: those eruptions can develop on both skin and/or mucosae (oral and genital). Oral involvement might also include xerostomia and taste change;

Psoriasis and rosacea: this agents might induce exacerbation or new onset of psoriasis;

Auto-immune bullous diseases: the development of auto-immune blistering disorders, especially bullous pemphigoid, have been reported.

Sarcoidosis: also reports of sarcoidosis (new onset or reactivation) are being recently published.

Vitiligo: immunotherapy has been linked to vitiligo-like lesions, mainly but not only in melanoma treated patients.^64^

A point of discussion is about whether or not the use of corticosteroids may antagonize the efficacy of immunotherapy. Current clinical data is limited and controversial. In some retrospective analyses, it was not associated with inferior responses to the oncologic treatment. A recent systematic review concluded that it “may not necessarily lead to poorer clinical outcomes”.^77^ On the other hand, retrospective analysis on lung cancer patients using corticosteroids at baseline or for irAE suggested a possible deleterious effect.^78^, ^79^ Most of the current guidelines and expert panels do not contra-indicate its use.^7^, ^10^, ^11^, ^12^, ^13^ As there are no definitive conclusions, we suggest that corticosteroids should be used with caution and always discussing its use with the rest of the team, especially the oncologists. For steroid-refractory cases, other immunomodulatory agents such as mycophenolate, infliximab, methotrexate and others, and also rituximab for blistering diseases might be necessary and a few reports with their use have been published.

## Final considerations

Dermatological adverse events are one of the most frequently observed toxicities from cancer treatments. Even though they rarely appear as life-threatening manifestations, they can lead to dose reductions or even discontinuation of oncologic therapy, interfering with disease outcome. In addition, they have a great impact in patient's quality of life. Being able to recognize and manage those skin-related toxicities gives dermatologists an important role on the multidisciplinary team, fundamental for the best supportive care of cancer patients.

Larger prospective randomized trials focusing on the management of the dermatological adverse events are still needed, but with the increasing development and recognition of the field of oncodermatology, this reality is each day closer.

## Financial support

Galderma.

## Authors’ contribution

Jade Cury Martins Approval of the final version of the manuscript; conception and planning of the study; elaboration and writing of the manuscript; obtaining, analysis, and interpretation of the data; effective participation in research orientation; intellectual participation in the propaedeutic and/or therapeutic conduct of the studied cases; critical review of the manuscript.

Adriana Pessoa Mendes Eris: Approval of the final version of the manuscript; conception and planning of the study; obtaining, analysis, and interpretation of the data; effective participation in research orientation; intellectual participation in the propaedeutic and/or therapeutic conduct of the studied cases; critical review of the manuscript.

Cristina Martinez Zugaib Abdalla: Approval of the final version of the manuscript; conception and planning of the study; obtaining, analysis, and interpretation of the data; effective participation in research orientation; intellectual participation in the propaedeutic and/or therapeutic conduct of the studied cases; critical review of the manuscript.

Giselle de Barros Silva: Approval of the final version of the manuscript; conception and planning of the study; obtaining, analysis, and interpretation of the data; effective participation in research orientation; intellectual participation in the propaedeutic and/or therapeutic conduct of the studied cases; critical review of the manuscript.

Veronica Paula Torel de Moura: Approval of the final version of the manuscript; conception and planning of the study; obtaining, analysis, and interpretation of the data; effective participation in research orientation; intellectual participation in the propaedeutic and/or therapeutic conduct of the studied cases; critical review of the manuscript.

Jose Antonio Sanches: Approval of the final version of the manuscript; conception and planning of the study; obtaining, analysis, and interpretation of the data; effective participation in research orientation; intellectual participation in the propaedeutic and/or therapeutic conduct of the studied cases; critical review of the manuscript.

## Conflicts of interest

None declared.
